# Pika Population Density Is Associated with the Composition and Diversity of Gut Microbiota

**DOI:** 10.3389/fmicb.2016.00758

**Published:** 2016-05-18

**Authors:** Huan Li, Jiapeng Qu, Tongtong Li, Jiabao Li, Qiang Lin, Xiangzhen Li

**Affiliations:** ^1^Key Laboratory of Environmental and Applied Microbiology, Environmental Microbiology Key Laboratory of Sichuan Province, Chengdu Institute of Biology, Chinese Academy of SciencesChengdu, China; ^2^University of Chinese Academy of SciencesBeijing, China; ^3^Key Laboratory of Adaptation and Evolution of Plateau Biota, Northwest Institute of Plateau Biology, Chinese Academy of SciencesXining, Qinghai, China

**Keywords:** host population density, plateau pika, gut microbiota, composition, diversity

## Abstract

Host population density is positively associated with the strength of social interactions or the frequency of physical contacts, and thus potentially influences microbial transmission among individuals. However, the relationship between host density and gut microbiota remains unknown. Here, we characterized the gut microbiota of plateau pikas (*Ochotona curzoniae*) in wild experimental fields with different host population densities. The abundance of some gut microbes significantly correlated with host density, such as Ruminococcaceae, Lachnospiraceae, and Staphylococcaceae. Intriguingly, host density was positively correlated with alpha diversity (Shannon diversity and observed species) of gut microbial communities. The inter-individual gut microbiota within high-density groups were more similar to each other than those of low-density groups. Host density significantly explained the variations in the gut microbiota, even after controlling sex, weight, diet and geographical locations. Based on the PICRUSt metagenome prediction, positive correlations were observed between host density and the relative abundances of 12 gene functions involved in cellular processes, environmental information processing and metabolism. These results indicate the importance of host density as a factor in shaping gut microbial composition and diversity in plateau pikas, and may further help us understand the social transmission of gut microbiota.

## Introduction

Metacommunity theory in ecology is often used to describe the dynamics and composition of species assembly within a given patch (environment or habitat). In this framework, the neutral processes of species colonization, extinction and drift lead to the differentiation of communities across patches (Hubbell, 2001). Two patches that are close to each other may exhibit more similar communities due to the species colonization more frequently based on the higher probability of repeated species migration and re-colonization. When the metacommunity theory is applied to gut microbial communities, each host individual can be regarded as one patch. In this case, gut microbial communities are expected to be more similar among individuals in habitats with higher population densities, because the individuals from these habitats have more chances to come into contact with each other, and frequent contact may promote the horizontal transmission of microbial communities.

From the perspective of social evolution, social interactions can mediate exposure and susceptibility to bacteria, and socially mediated transmission is able to affect the evolutionary costs and benefits of social relationships (Lombardo, 2008; Archie and Theis, 2011). However, the influence of social relationships on the gut microbiota is an underappreciated outcome of group living, and is associated with disease transmission (Garrett et al., 2010; Henao-Mejia et al., 2012) and defense against pathogens (Koch and Schmid-Hempel, 2011). For instance, co-housing mice in the laboratory promotes the horizontal transmission of gut bacteria associated with obesity (Henao-Mejia et al., 2012) and inflammatory bowel disease (Garrett et al., 2010), indicating that the social interactions may increase the microbiota-associated disease risk. Moreover, socially transmitted bacteria protect bumblebees against a widespread and highly virulent gut parasite (*Crithidia bombi*) (Koch and Schmid-Hempel, 2011), suggesting that microbial transmission can provide some benefits for the host. It has been shown that host density was a determining factor of abundance in parasite communities, and that transmission rates of parasites rely on host population density (Arneberg et al., 1998). Similarly, the probability of microbial transmission through social interactions should depend closely on host population density in a limited space. A recent report showed that social interactions predict gut microbiome composition in wild baboons (Tung et al., 2015). However, measuring social interactions in the wild is quite difficult, potentially inaccurate and time-consuming. As an alternative, host population density is easy to measure and is associated with social interactions (Wang et al., 2005); thus it may potentially influence the composition of gut microbiota. Yet, to date, the relationship between host density and gut microbiota remains unknown.

Plateau pika (*Ochotona curzoniae*) is a keystone species for biodiversity and one of the most important animals shaping the landscape and function of grassland ecosystems in the Qinghai-Tibet Plateau (Smith and Foggin, 1999; Li et al., 2013). They are highly social herbivorous mammals that live in family groups (Dobson et al., 1998). Under moderate population density, they create microhabitats that result in an increase in plant species diversity and contribute positively to ecosystem-level dynamics (Smith and Foggin, 1999). Plateau pikas could reach a very high population density in the Qinghai-Tibet Plateau, especially in the degraded grassland. A high level of population density may also increase the transmission of gut microbes, and the risk of microbiota-associated diseases. However, it remains unknown whether pika population density is associated with gut microbiota. Here, we used plateau pikas as an animal model to explore whether host density is correlated with the composition and diversity of gut microbiota. Our results showed that host density is positively correlated with alpha diversity of gut microbiota, and the inter-individual microbiota within the high-density groups were more similar to each other than those within the low-density groups.

## Materials and methods

### Experimental design and sample collection

**Figure 1:**
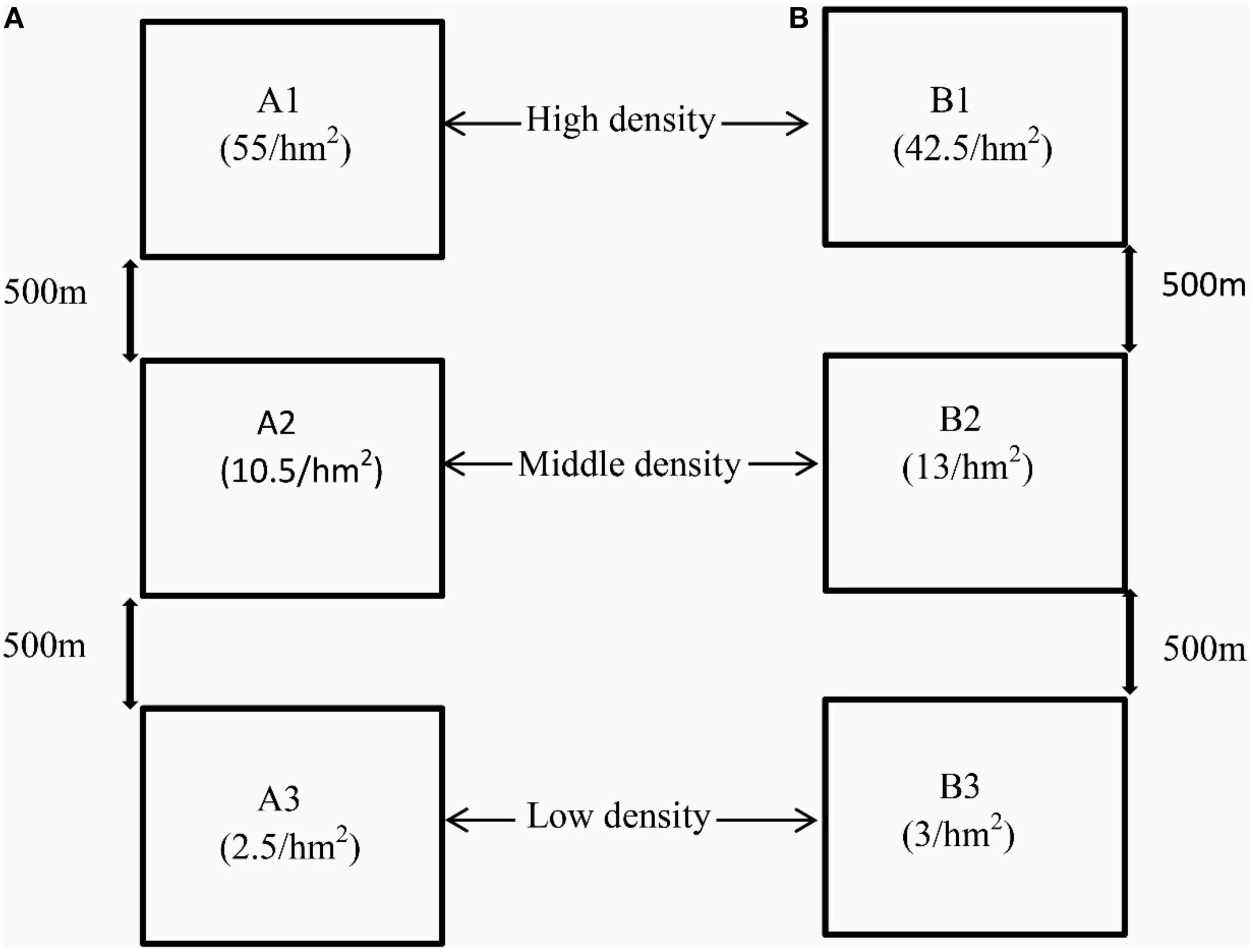
**The experimental design in this study**. A total of six plots from two experimental fields were selected. The geographical distance is 80 kilometers between two sites **(A, B)**. The population densities of six plots, A1, A2, A3, B1, B2, and B3, were 55, 10.5, 2.5, 42.5, 13, and 3 individuals per hm^2^, respectively. All the plots can be divided into three groups, including high-density groups (A1 and B1), middle-density groups (A2 and B2), and low-density groups (A3 and B3).

We chose two experimental fields (A and B) that harbored homogeneous grass environments and very similar plant communities. They were located in the near Zeku County of the Qinghai-Tibet Plateau. Due to the different extent of the killings by humans and the population migration of plateau pikas in the past few years, different population densities were naturally formed in these experimental sites. The geographical distance between A and B is about 80 kilometers. Each experimental field consisted of three 1 × 1 km plots. To reduce artificial disturbance, each plot was surrounded by a metal fence. Based on our long-term observations, there were almost no natural enemies of plateau pikas in these plots. The distance between the two plots in the same field was at least 500 m. The pika population density in each plot was measured based on the walked transect method (Qu et al., 2011). The population densities (individuals per hm^2^) of the six plots A1, A2, A3, B1, B2, and B3 were 55, 10.5, 2.5, 42.5, 13, and 3, respectively (Figure 1). In order to explore the possible differences of food resources for herbivorous pikas between fields A and B, the plant species were identified based on morphological characteristics, and the plant cover was measured in each experimental field according to the method described by Hogan (2010).

All animal care procedures were consistent with the provision of the Institution of Animal Care and the Ethics Committee of Chengdu Institute of Biology, Chinese Academy of Sciences. Processing of wild animals and sample collection were strictly in accordance with the guidelines of our academic institutions. We randomly captured 7–9 individuals with ropes in each plot. After pikas were euthanized and dissected, their gut contents were immediately collected from the caecum of wild pikas, and stored immediately at −20°C in a portable refrigerator. A total of 50 caecum content samples were collected from the six plots, including A1 (*n* = 7), A2 (*n* = 8), A3 (*n* = 9), B1 (*n* = 9), B2 (*n* = 9), and B3 (*n* = 8). The corresponding information of each sample was listed in Table S1.

### 16S rRNA sequencing and bioinformatics analysis

The Ezup genomic DNA extraction kit for soil (Sangon Biotech, China) was used to extract total DNA of gut contents. DNA concentration and purity were determined using the Nanodrop 2000 Spectrophotometer. The extracted DNA was diluted to 10 ng/μL for PCR amplification. In order to amplify the V4–V5 hypervariable region of the microbial 16S rRNA gene, the universal primers 515F (5′-GTGYCAGCMGCCGCGGTA-3′) and 909R (5′-CCCCGYCAATTCMTTTRAGT-3′) with 12 nt unique barcode at 5′-end of 515F (Tamaki et al., 2011) were used. PCR amplifications were performed in duplicate with 25 μL reaction mix containing 1 × PCR buffer, 1.5 mM MgCl_2_, each deoxynucleoside triphosphate at 0.2 mM, each primer at 1.0 μM and 0.25 U of Ex Taq (TaKaRa, Dalian) and 10 ng genomic DNA. The thermal cycling procedure consisted of an initial denaturation step at 94°C for 3 min, followed by 30 cycles of 94°C for 40 s, 56°C for 60 s, and 72°C for 60 s, and a final extension at 72°C for 10 min. After PCR amplification, the two PCR products were mingled together and subjected to electrophoresis using 1.2% agarose gel. The correct band (~400 bp) was excised and purified using SanPrep DNA Gel Extraction Kit (Sangon Biotech, China) and quantified with the Nanodrop 2000 Spectrophotometer. All samples were pooled together with an equal molar amount from each sample. The sequencing samples were prepared using the TruSeq DNA kit according to the manufacturer's instruction. The purified library was diluted, denatured, re-diluted, mixed with PhiX (equal to 30% of final DNA amount), as described in the Illumina library preparation protocols, and then applied to an Illumina Miseq platform for sequencing (Reagent Kit V2) at the Environmental Genome Platform of Chengdu Institute of Biology.

The raw sequence data were processed using QIIME Pipeline-Version 1.7.0 (http://qiime.org/tutorials/tutorial.html). All sequences were trimmed and assigned to each sample based on their barcodes (barcode mismatches = 0). The overlapping paired-end reads were merged using the FLASH-1.2.8 software (Magoc and Salzberg, 2011). The merged sequences with high quality (reads length >300 bp, without ambiguous base “N,” and average base quality score >30) were used for further analysis. Due to possible contamination of chloroplast sequences in PCR amplification, we employed the Metaxa2 software tool to remove chloroplast sequences from our large sequencing datasets (Bengtsson-Palme et al., 2015). The aligned 16S rRNA gene sequences were used for a chimera check using the Uchime algorithm (Edgar et al., 2011). All sequences were clustered into operational taxonomic units (OTUs) at a 97% identity threshold using CD-HIT (Li and Godzik, 2006). Singleton OTUs were filtered out. To compare samples with different sequencing depths, each sample was rarefied to the same number of reads (8234 sequences). The alpha diversity indices, including observed species and Shannon diversity, were calculated. To assess beta diversity, the unweighted and weighted UniFrac distance metrics, which use phylogenetic information to calculate community similarity (Lozupone and Knight, 2005), were produced through the QIIME pipeline. In addition, in order to compare community similarity based on the presence/absence of OTUs, the Jaccard index was used; to compare community similarity based on OTU abundance, the Bray-Curtis dissimilarity matric was used with Hellinger transformed data (Dixon, 2003). The rarefaction curves were generated from the Goods coverage at the OTU level. Taxonomy was assigned using the Ribosomal Database Project classifier (Wang et al., 2007).

The original sequence data are available at the European Nucleotide Archive by accession number PRJEB11203 (http://www.ebi.ac.uk/ena/data/view/PRJEB11203).

### Statistical analysis

Because the sample size was uneven in each group, an analysis of similarity (ANOSIM), which does not allow for interaction terms (Dill-McFarland et al., 2015), was applied to evaluate if microbial communities were significantly different across groups. ANOSIM analysis was implemented using the procedure “anosim” in the R “vegan” package (Warton et al., 2012). We calculated the average weighted UniFrac dissimilarity within each group. In order to explore the relationship between host density and the average weighted UniFrac dissimilarity, the Spearman correlation analysis was tested. Differences in the levels of variation within each group were tested with permutational tests of multivariate dispersions (PERMDISP). The Spearman correlations between the mean relative abundance (>0.05%) of microbial taxa at phylum, family and genus levels, and host population density were analyzed using SPSS 13.0 software. False discovery rate (FDR) values were evaluated using the Benjamini-Yekutieli method (Benjamini and Yekutieli, 2001).

### Network analysis

In order to better understand the interactions among different species in the gut microbial communities and their correlations with the host population density, we constructed phylogenetic molecular ecological networks (pMENs) through Random Matrix Theory (RMT)-based methods, according to the molecular ecological network analyses pipeline (http://ieg2.ou.edu/MENA/main.cgi) with default parameters based on the OTUs from the 16S rRNA gene sequences. This approach is automatically defined and robust to noise, thus it provides remarkable solutions to several common issues associated with high-throughput metagenomic data (Deng et al., 2012). Only those OTUs identified in more than half of all individuals were used for network construction. The OTU modules were detected by fast greedy modularity optimization. The modules with more than 6 nodes were used for network-Eigengene analysis. The module-EigenGene analysis was then used to detect the correlations between microbial modules and host density. Cytoscape 3.0.2 software was used to visualize the final ecological network.

### Predicted metagenomes

PICRUSTv1.0.0 (Langille et al., 2013) was used to predict the abundances of KOs from OTU abundances rarefied at 8234 reads per sample. We calculated the mean relative abundance of gene functions in each group. Thereafter, the spearman correlation between population density and predicted KEEG functions was analyzed using SPSS 13.0 software. *P*-values have been corrected using the false discovery rate control.

## Results

### Diversity of gut microbiota in plateau pikas

To compare samples with different sequencing depths, each sample was rarefied to 8234 sequences. At a threshold of 97% sequence identity, 37,258 unique OTUs were identified in all samples. The OTU-level rarefaction curve of Goods coverage across all samples has reached stable values (Figure S1), showing that most of the gut microbial diversity had already been captured in our study, despite the possibility of detecting rare new OTUs with an additional sequencing depth.

**Figure 2:**
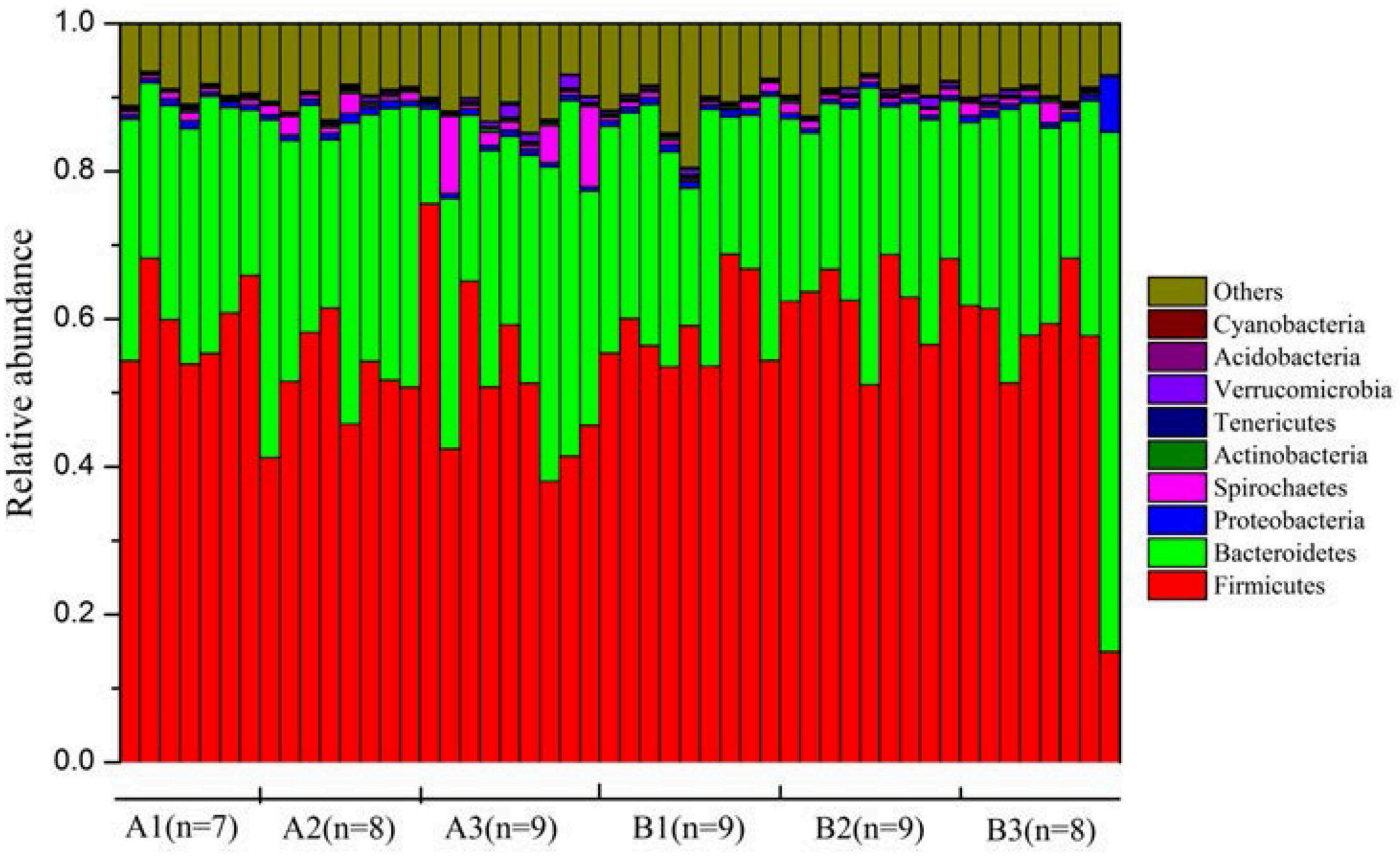
**The composition of pika gut microbiota at phylum level**. Only those phyla with the relative abundances >0.1% were shown.

Plateau pikas harbored diverse gut microbial communities. Across all samples, 90.1 and 47.2% of total sequences were assigned to 29 phyla and 147 families, respectively. Firmicutes (mean ± SEM = 56.5% ± 1.4%), Bacteroidetes (30.2% ± 1.3%), Spirochaetes (1.3% ± 0.3%), and Proteobacteria (0.9 % ± 0.1%) were the dominant phyla across all samples. The composition of each sample was visualized at the phylum level in Figure 2. At the family level, the gut microbiota of the plateau pikas was dominated by Ruminococcaceae (18.1% ± 0.7%), S24-7 (13.4% ± 1.2%), Prevotellaceae (7.4% ± 0.5%) and Lachnospiraceae (2.8% ± 0.1%). However, only a small proportion of sequences (19.3 %) were identified at the genus level. The dominant genera were *Prevotella* (7.2% ± 0.5%), *Oscillospira* (5.1% ± 0.3%), *Ruminococcus* (2.1% ± 0.1%), and *Treponema* (1.1% ± 0.3%). The compositions of gut microbiota at phylum, family and genus levels were provided in Table S2.

### Population density is associated with the composition of pika gut microbiota

The Spearman correlation analysis was implemented to investigate the relationships between the relative abundances of major taxa (phyla, families, and genera) and host population density. At the phylum level, Acidobacteria were positively correlated with the host density, while Spirochaetes showed negative correlations with the host density. At the family level, Ruminococcaceae, Lachnospiraceae, and Staphylococcaceae were positively associated with population density, while Spirochaetaceae, Coriobacteriaceae, and Clostridiaceae showed negative correlations with the host density. At the genus level, *Oscillospira, Roseburia*, and *Clostridium* showed positive correlations with the host population density, whereas *Treponema* showed negative correlations with the host density (Table 1).

**Table 1:** **Gut microbial taxa significantly correlated with pika population density**.

	***r***	***P*-value**
**PHYLA**		
Spirochaetes	−0.943	0.005^**^
Acidobacteria	0.943	0.005^**^
**FAMILIES**		
Ruminococcaceae	0.829	0.042^*^
Lachnospiraceae	0.886	0.019^*^
Spirochaetaceae	−0.943	0.005^**^
Coriobacteriaceae	−0.943	0.005^**^
Clostridiaceae	−0.886	0.019^*^
Staphylococcaceae	0.886	0.019^*^
**GENERA**		
*Oscillospira*	0.829	0.042^*^
*Treponema*	−0.943	0.005^**^
*Roseburia*	0.829	0.042^*^
*Clostridium*	0.986	0.0003^***^

Spearman correlation analysis is used. P-value has been corrected using the false discovery rate control. The significance is indicated by

* P < 0.05,

** P < 0.01,

*** *P < 0.001*.

### Population density is associated with the diversity of gut microbiota

The alpha diversity of pika gut microbiota from different groups was calculated (Table 2). Both the Shannon diversity (Spearman rho = 0.87, *P* < 0.05) and observed species (Spearman rho = 0.829, *P* < 0.05) showed significant positive associations with the host density.

**Table 2:** **The alpha diversity of pika gut microbiota in different experimental plots and Spearman correlations with host population densities**.

**Experimental plots (population density: individuals/hm^2^)**	**Shannon diversity**	**Observed species**
A1(55)	10.7 ± 0.1ab	3337 ± 103ab
A2(10.5)	10.6 ± 0.1ab	3330 ± 112ab
A3(2.5)	10.5 ± 0.13ab	3147 ± 91ab
B1(42.5)	10.8 ± 0.04a	3433 ± 23a
B2(13)	10.6 ± 0.1ab	3299 ± 73ab
B3(3)	10.4 ± 0.23b	3105 ± 204b
Spearman's correlation coefficients	0.87^*^	0.829^*^

Data are expressed as mean ± SEM. Different letters indicate significant differences of alpha diversity among experimental plots. The significant correlation is indicated by

* *P < 0.05*.

**Figure 3:**
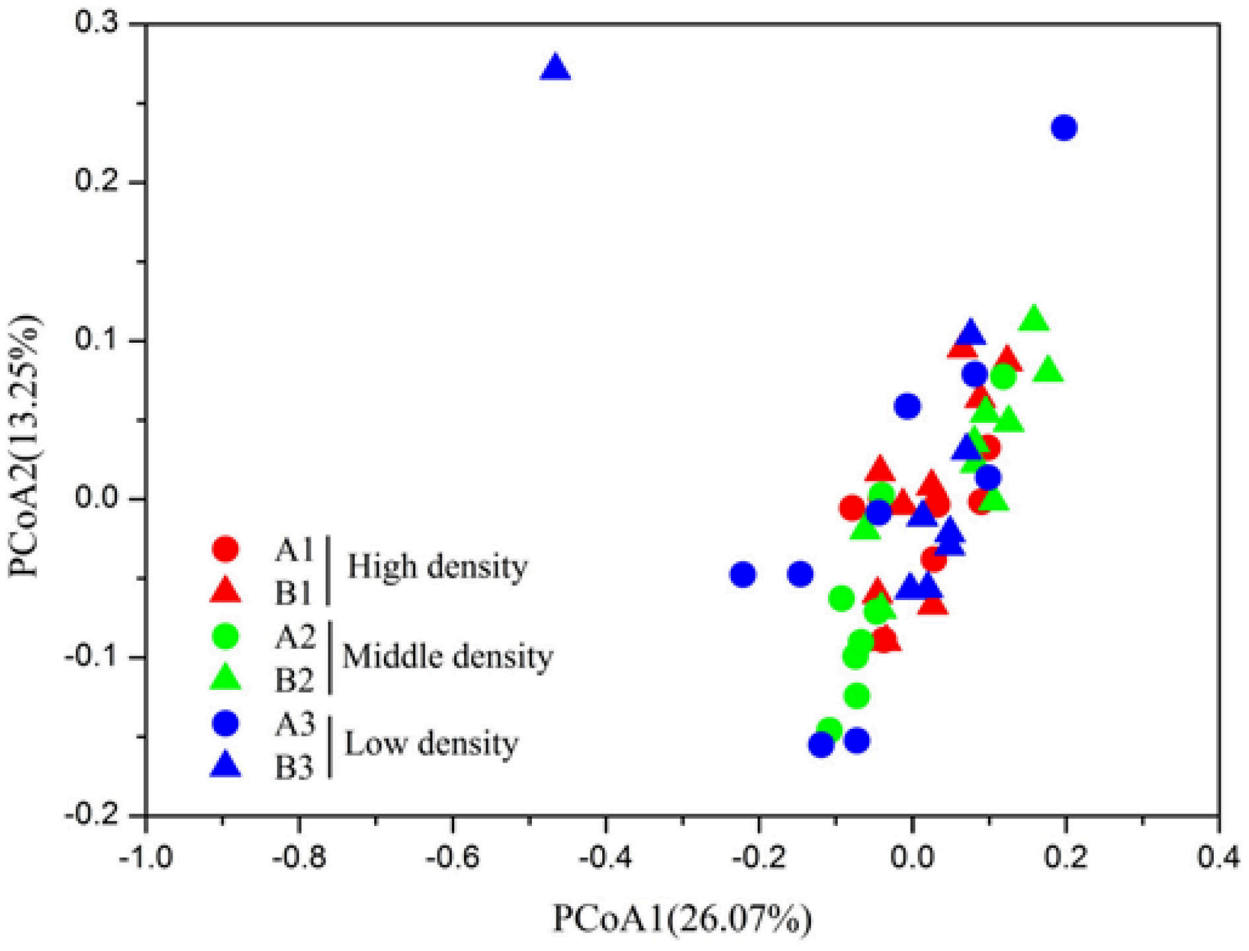
**The Principal coordinate analysis of pika gut microbiota of high-density groups (A1 and B1), middle-density groups (A2 and B2), and low-density groups (A3 and B3)**.

**Figure 4:**
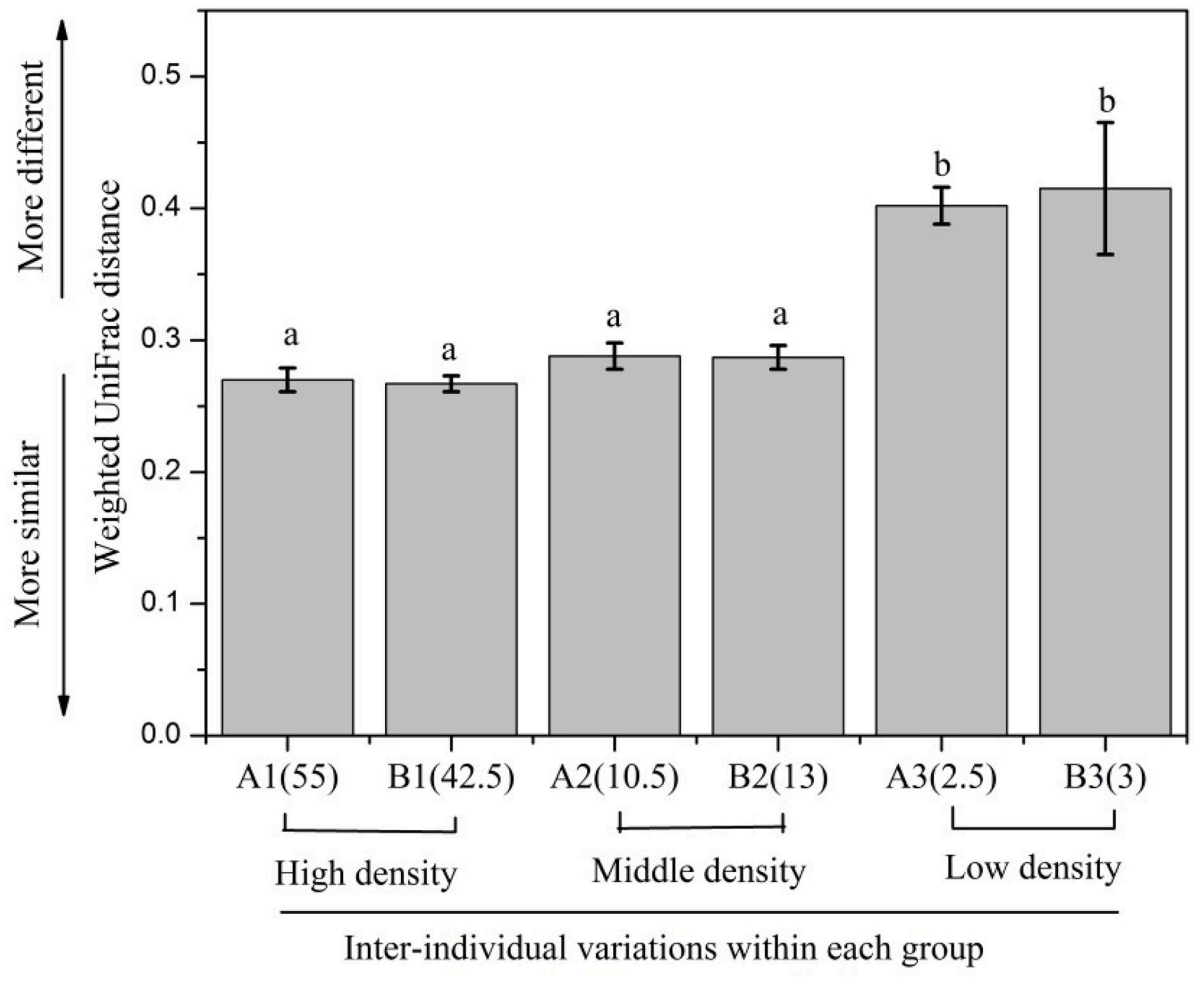
**The inter-individual variations of gut microbiota within each group**. The significance is indicated by different letters (*P* < 0.001, one-way ANOVA).

In order to evaluate the beta diversity of pika gut microbiota, we calculated the Jaccard, Bray-Curtis, unweighted UniFrac and weighted UniFrac distance metrics across samples. The results using these distance metrics were very similar; thus, only the results for the weighted UniFrac were presented here. The gut microbiota in pikas were significantly influenced by the host density (ANOSIM, *r* = 0.2, *P* < 0.05). The microbial communities within the high-density groups (A3 and B3) or middle-density groups (A2 and B2) had less inter-individual variations than those of the low-density groups (A1 and B1). That is, the individuals within the high-density groups or middle-density groups clustered more tightly together than those within the low-density groups, Figure 3). The results were confirmed by PERMDISP (*P* < 0.001). In addition, we calculated the average weighted UniFrac dissimilarities of gut microbiota within each plot (Figure 4). The inter-individual gut microbiota within the high-density groups or middle-density groups were more similar to each other than those within the low-density groups (One-way ANOVA, *P* < 0.001). Host density was negatively correlated with the average inter-individual dissimilarity within each plot (Spearman rho = −0.886, *P* < 0.05). Notably, there was one outlier sample shown in Figure 3. However, we still obtained similar results even after removing this sample.

### Differences of gut microbial compositions among different population densities were unlikely explained by diet

Previous research showed that diet played an important role in shaping the differences of gut microbial compositions in primates and humans (Degnan et al., 2012; Yatsunenko et al., 2012). However, the two experimental fields in this study harbored a very homogeneous environment and similar plant communities. About 50% of plant coverage in the experimental plot A or B was contributed by *Kobresia humilis*, and similar proportions were contributed by other plant species, such as *Ajuga lupulina, Leontopodium nanum*, and *Potentilla anserine* (Figure S2). Only three plant species (*Stipa capillata, Lagotis Gaertn*, and *Aconitum szechenyianum*) were different between plots A and B (Table S3). Moreover, we calculated the effects of host density, diet diversity, body weight, geographical locations (A and B) and sex in shaping the gut microbiota of plateau pikas (Table S4). The results showed that only host density significantly influenced the gut microbial composition (ANOSIM, *r* = 0.2, *P* < 0.05), while diet diversity, host weight, sex and geographical locations did not show significant effects (ANOSIM, *P* > 0.05, Table S4).

### Network analysis showed that density positive-correlation microbes were mainly *Prevotella, Ruminococcus, and Oscillospira*

**Figure 5:**
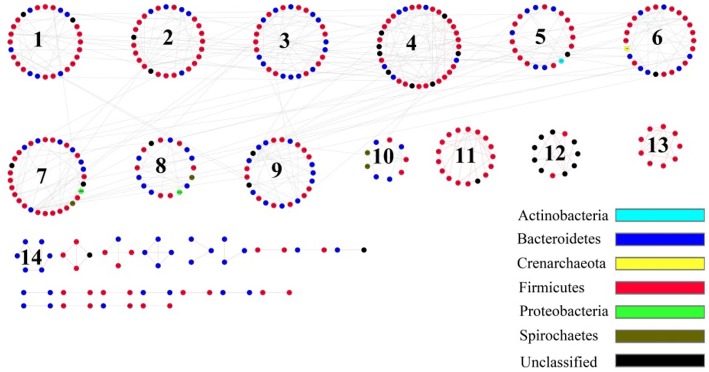
**The Molecular Ecological Networks (MEN) of pika gut microbial communities**. Number in the circles is module number. Red line means positive correlation, and gray line means negative correlation.

Network analysis can reveal microbial interactions of different species in a microbial community, such as competition and mutualism. The species that own a similar niche or function form a module (Deng et al., 2012). Based on network analysis, the gut microbiota of plateau pikas consisted of 14 modules (Figure 5). Using module-EigenGene analysis, we found that the module 10, which mainly consisted of OTUs affiliated with Clostridiales, was negatively associated with host density, whereas modules 2, 3, 4, and 9, which mainly contained OTUs associated with *Prevotella, Ruminococcus*, and *Oscillospira*, showed positive correlations with host density (Table S5). The taxonomy of all the OTUs from each module was listed in Table S6.

### Population density is associated with gene functions of gut microbiota

Correlations between host density and gene functions based on the PICRUSt metagenome prediction were tested. The relative abundances of 12 gene functions involved in cellular processes, environmental information processing and metabolism were positively correlated with host density (Table S7). In particular, those genes involved in glycan biosynthesis and metabolism, such as N-glycan biosynthesis, glycosaminoglycan degradation and other glycan degradation, were positively associated with host density (*P* < 0.05).

## Discussion

Our results provided robust evidence that host population density is associated with the composition and diversity of gut microbiota in wild plateau pikas. To our knowledge, this is the first report to demonstrate that the host density can explain the variations in gut microbial communities after excluding the effects of sex, weight, diet, and geographical location. Host density was positively correlated with the strength of social interactions or the frequency of physical contacts. Thus, our results indicated that physical contacts or social relationships may play important roles in shaping gut microbial composition and diversity in natural populations.

Microbial transmission is very important in maintaining the diversity of the gut microbiota in wild mammals (Lombardo, 2008). Similar to mice and bumblebees (Garrett et al., 2010; Koch and Schmid-Hempel, 2011), plateau pikas are also coprophagic, thus a higher host density may increase the probability and incidences of microbial fecal-oral transfers among individuals. In addition to fecal-oral transfer, the proximity and physical contact between hosts was associated with the exchange of gut microbiota within the host species (Yatsunenko et al., 2012; VanderWaal et al., 2014). In experimental mice, hosts reared in the same cage are inclined to share their gut microbes (Campbell et al., 2012). In wild baboons, closer grooming partners harbor more similar gut microbial communities (Tung et al., 2015). For plateau pikas, the frequency of affiliative behaviors (e.g., kissing and contact) is closely correlated with host density (Wang et al., 2005), and potentially influences the transfer frequency from hand to mouth and from mouth to mouth. Social microbial transfer is not limited to the same animal species, but also occurs between different species. In addition to direct transmission, the microbial transmission can be indirectly mediated through shared environments, such as soil (Nunn et al., 2011).

Previous research on social-mediated transmission focuses on parasites (Nunn et al., 2011) or pathogens (Fournier et al., 2015), which may exert diseases or adverse effects on hosts. However, this study implicated that social transmission microbes may also provide powerful benefits for the host. In our study, the relative abundances of Ruminococcaceae and Lachnospiraceae were positively correlated with host density, and these density positive-correlation bacteria were likely social transmission microbes. Many members of these families harbor various genes encoding cellulase, hemicellulase or oligosaccharide-degrading enzymes (Dai et al., 2014); thus, they may play important roles in plant polysaccharide degradation. In particular, the genus *Roseburia*, one common butyrate producer (Duncan, 2002), showed positive correlations with host density, implicating that SCFA-producing bacteria can also be transmitted among pika populations. Intriguingly, the dominant genus *Oscillospira*, which was associated with fresh forage diets (Mackie et al., 2003), also showed a positive correlation with host density. Plateau pikas often consume a large amount of fresh grass in summer for their development and reproduction. When the host density increases in a limited space, the plateau pikas may compete for fresh grass more strongly, thus, increase the abundance of *Oscillospira* associated with fresh diets. In this study, metagenome prediction based on PICRUSt showed the relative abundances of function genes involved in glycan biosynthesis and metabolism were positively correlated with host density, indicating that host density may indirectly influence the function of gut microbiota. Future work may address the relationship between gut microbiota function and host population density in order to understand overall functional ecology of microbial transmission.

Some bacteria, such as *Treponema*, were negatively correlated with host density. However, these bacteria may have similar spatial and nutritional niches with those density positive-correlation microbes. Their abundances may be strongly influenced by the density positive-correlation microbes through microbial interactions. Understanding the function of these density negative-correlation microbes has important implications for the study of host health and nutrition. On the one hand, some members of the genus *Treponema* may cause mammalian diseases, although they are considered as nonpathogenic members in the gut community of ruminants and nonhuman primates (Stanton and Canale-Parola, 1979; McKenna et al., 2008). Future research is needed to explore whether these microbes are associated with the occurrence of pika disease. On the other hand, some species in this genus are associated with the digestion of specific soluble fibers (Bekele et al., 2011), and facilitate the cellulose degradation with co-occurring bacteria (Kudo et al., 1987).

The assumption that host density is associated with the alpha diversity of gut microbiota by positively affecting microbial transmission rates is crucial in epidemiological theory. We can infer that the transmission rates of microbes are accelerated from a minority of individuals to a wider range of host individuals in the high-density groups. As diversity is important for the stability and performance of all ecosystems (Clarke et al., 2014), the positive correlations between host density and alpha diversity of gut microbiota suggest that social microbial transmission may improve stability and performance of gut microbiota in high-density groups. Social transmission microbes in a high-density group may be more important to regulate inter-individual differences of gut microbiota. The hypothesis is consistent with our results that the inter-individual microbial compositions within high-density groups were more similar to each other when compared to those within low-density groups. Our findings supported that gut microbiota can be regarded as a result of “supply-side” ecology (Lewin, 1986); that is, you get what you are exposed to Bush (1990). Host density is an important determinant of exposure rates (Arneberg et al., 1998); thus higher microbial diversity and less inter-individual variations of gut microbiota in high-density groups are the results of higher exposure rates. From the view of the metacommunity theory, plateau pikas can be regarded as free-living animals occupying a set of discrete patches, and patch occupancy (i.e., prevalence of microbial species) often increases with patch density. It seems that low-density patches are less frequently occupied because they are less likely to come in contact with other pikas/microbes. Theoretically, the connection is formed by host density positively affecting microbial transmission rates. When host densities increase, those exposed bacteria (e.g., feces-associated bacteria or skin-associated bacteria) have more chances to contact with other host individuals.

Here, we highlighted the effects of transmission rates on gut microbiota, and considered contact rates as a sole correlation parameter of host density. However, we can't ignore the possible influences of other factors relevant to microbial transmission, such as host family size, kinship relationship, sociality and population structure. It remains uncertain whether population density has a widespread effect on microbial diversity in other host species. It would be particularly valuable for understanding the linkage between microbes-associated disease risk and host population density.

## Author contributions

HL, JQ designed the experiments. HL, JQ, TL, and QL performed the field experiments. HL, XL wrote the paper. JL revised the paper.

### Conflict of interest statement

The authors declare that the research was conducted in the absence of any commercial or financial relationships that could be construed as a potential conflict of interest.

## References

[B1] Archie E. A. Theis K. R. (2011). Animal behaviour meets microbial ecology. Anim. Behav. 82, 425–436. 10.1016/j.anbehav.2011.05.029

[B2] Arneberg P. Skorping A. Grenfell B. Read A. F. (1998). Host densities as determinants of abundance in parasite communities. Proc Biol. Sci. 265, 1283–1289. 10.1098/rspb.1998.0431

[B3] Bekele A. Z. Koike S. Kobayashi Y. (2011). Phylogenetic diversity and dietary association of rumen Treponema revealed using group-specific 16S rRNA gene-based analysis. FEMS Microbiol. Lett. 316, 51–60. 10.1111/j.1574-6968.2010.02191.x 21204927

[B4] Bengtsson-Palme J. Hartmann M. Eriksson K. M. Pal C. Thorell K. Larsson D. G.. (2015). Metaxa2: improved identification and taxonomic classification of small and large subunit rRNA in metagenomic data. Mol. Ecol. Resour. 15, 1403–1414. 10.1111/1755-0998.12399 25732605

[B5] Benjamini B. Y. Yekutieli D. (2001). The control of the false discovery rate in multiple testing under dependency. Ann. Stat. 473, 174–180. 10.1186/1471-2105-9-114

[B6] Bush A. O. (1990). Helminth communities in avian hosts: determinants of pattern, in Parasite Communities: Patterns and Processes, eds Esch G. Bush A. O. Aho J. M. (London: Chapman and Hall), 197–232.

[B7] Campbell J. H. Foster C. M. Vishnivetskaya T. Campbell A. G. Yang Z. K. Wymore A.. (2012). Host genetic and environmental effects on mouse intestinal microbiota. ISME J. 6, 2033–2044. 10.1038/ismej.2012.54 22695862 PMC3475380

[B8] Clarke S. F. Murphy E. F. O'Sullivan O. Lucey A. J. Humphreys M. Hogan A.. (2014). Exercise and associated dietary extremes impact on gut microbial diversity. Gut 63, 1913–1920. 10.1136/gutjnl-2013-306541 25021423

[B9] Dai X. Tian Y. Li J. Su X. Wang X. Zhao S.. (2014). Metatranscriptomic analyses of plant cell wall polysaccharide degradation by microorganisms in cow rumen. Appl. Environ. Microbiol. 81, 1375–1386. 10.1128/AEM.03682-14 25501482 PMC4309707

[B10] Degnan P. H. Pusey A. E. Lonsdorf E. V. Goodalld J. Wroblewski E. E. Wilson M. L.. (2012). Factors associated with the diversification of the gut microbial communities within chimpanzees from Gombe National Park. Proc. Natl. Acad. Sci. U. S. A. 109, 13034–13039. 10.1073/pnas.1110994109 22826227 PMC3420156

[B11] Deng Y. Jiang Y. H. Yang Y. He Z. Luo F. Zhou J. (2012). Molecular ecological network analyses. BMC Bioinformatics 13:113. 10.1186/1471-2105-13-113 22646978 PMC3428680

[B12] Dill-McFarland K. A. Weimer P. J. Pauli J. N. Peery M. Z. Suen G. (2015). Diet specialization selects for an unusual and simplified gut microbiota in two- and three-toed sloths. Environ. Microbiol. 18, 1391–1402. 10.1111/1462-2920.13022 26271635

[B13] Dixon P. (2003). VEGAN, a package of R functions for community ecology. J. Veg. Sci. 14, 927–930. 10.1111/j.1654-1103.2003.tb02228.x

[B14] Dobson F. S. Smith A. T. Wang X. G. (1998). Social and ecological influences on dispersal and philopatry in the plateau pika (*Ochotona curzoniae*). Behav. Ecol. 9, 622–635. 10.1007/s10344-011-0574-2

[B15] Duncan S. H. (2002). Roseburia intestinalis sp. nov., a novel saccharolytic, butyrate-producing bacterium from human faeces. Int. J. Syst. Evol. Microbiol. 52, 1615–1620. 10.1099/ijs.0.02143-0 12361264

[B16] Edgar R. C. Haas B. J. Clemente J. C. Quince C. Knight R. (2011). UCHIME improves sensitivity and speed of chimera detection. Bioinformatics 27, 2194–2200. 10.1093/bioinformatics/btr381 21700674 PMC3150044

[B17] Fournier A. Young I. Rajic A. Greig J. LeJeune J. (2015). Social and economic aspects of the transmission of pathogenic bacteria between wildlife and food animals: a thematic analysis of published research knowledge. Zoonoses Public Health 62, 417–428. 10.1111/zph.12179 25611914

[B18] Garrett W. S. Gallini C. A. Yatsunenko T. Michaud M. DuBois A. Delaney M. L.. (2010). Enterobacteriaceae act in concert with the gut microbiota to induce spontaneous and maternally transmitted colitis. Cell Host Microbe. 8, 292–300. 10.1016/j.chom.2010.08.004 20833380 PMC2952357

[B19] Henao-Mejia J. Elinav E. Jin C. Hao L. Mehal W. Z. Strowig T.. (2012). Inflammasome-mediated dysbiosis regulates progression of NAFLD and obesity. Nature 482, 179–185. 10.1038/nature10809 22297845 PMC3276682

[B20] Hogan B. (2010). The Plateau Pika: A Keystone Engineer on the Tibetan Plateau. Phoenix: Arizona State University.

[B21] Hubbell S. P. (2001). The Unified Neutral Theory of Biodiversity and Biogeography. Princeton, NJ: Princeton University Press.

[B22] Koch H. Schmid-Hempel P. (2011). Socially transmitted gut microbiota protect bumble bees against an intestinal parasite. Proc. Natl. Acad. Sci. U.S.A. 108, 19288–19292. 10.1073/pnas.1110474108 22084077 PMC3228419

[B23] Kudo H. Cheng K. Costerton J. (1987). Interactions between *Treponema bryantii* and cellulolytic bacteria in the *in vitro* degradation of straw cellulose. Can. J. Microbiol. 33, 244–248. 10.1139/m87-041 3567744

[B24] Langille M. G. Zaneveld J. Caporaso J. G. McDonald D. Knights D. Reyes J. A.. (2013). Predictive functional profiling of microbial communities using 16S rRNA marker gene sequences. Nat. biotech. 31, 814–821. 10.1038/nbt.2676 23975157 PMC3819121

[B25] Lewin R. (1986). Supply-side ecology. Science 234, 25–27. 10.1126/science.234.4772.25 17742631

[B26] Li N. Li H. Lei G. Zhou Y. (2013). Ecological function of plateau pika(*Ochotona curzoniae*). Chinese J. Wildl. 34, 238–242. 10.3969/j.issn.1000-0127.2013.04.013 (In Chinese).

[B27] Li W. Godzik A. (2006). Cd-hit: a fast program for clustering and comparing large sets of protein or nucleotide sequences. Bioinformatics 22, 1658–1659. 10.1093/bioinformatics/btl158 16731699

[B28] Lombardo M. P. (2008). Access to mutualistic endosymbiotic microbes: an underappreciated benefit of group living. Behav. Ecol. Sociobiol. 62, 479–497. 10.1007/s00265-007-0428-9

[B29] Lozupone C. Knight R. (2005). UniFrac: a new phylogenetic method for comparing microbial communities. Appl. Environ. Microbiol. 71, 8228–8235. 10.1128/AEM.71.12.8228-8235.2005 16332807 PMC1317376

[B30] Mackie R. I. Aminov R. I. Hu W. Klieve A. V. Ouwerkerk D. Sundset M. A.. (2003). Ecology of uncultivated oscillospira species in the rumen of cattle, sheep, and reindeer as assessed by microscopy and molecular approaches. Appl. Environ. Microbiol. 69, 6808–6815. 10.1128/aem.69.11.6808-6815.2003 14602644 PMC262257

[B31] Magoc T. Salzberg S. L. (2011). FLASH: fast length adjustment of short reads to improve genome assemblies. Bioinformatics 27, 2957–2963. 10.1093/bioinformatics/btr507 21903629 PMC3198573

[B32] McKenna P. Hoffmann C. Minkah N. Aye P. P. Lackner A. Liu Z.. (2008). The macaque gut microbiome in health, lentiviral infection, and chronic enterocolitis. PLoS Pathog 4:e20. 10.1371/journal.ppat.0040020 18248093 PMC2222957

[B33] Nunn C. L. Thrall P. H. Leendertz F. H. Boesch C. (2011). The spread of fecally transmitted parasites in socially-structured populations. PLoS ONE 6:e21677. 10.1371/journal.pone.0021677 21738763 PMC3128086

[B34] Qu J. Li W. Yang M. Li K. Zhang Y. (2011). Methods for large scale assessment of small mammal abundance in open habitats plateau pika (*Ochotona curzoniae*) in alpine grassland. Pol. J. Ecol. 59, 829–833. Available online at: http://yadda.icm.edu.pl/yadda/element/bwmeta1.element.baztech-article-BGPK-3624-3980?q=9a4b265c-b46c-4216-ab40-c447add79f4c$2&qt=IN_PAGE

[B35] Smith A. T. Foggin J. M. (1999). The plateau pika (*Ochotona curzoniae*) is a keystone species for biodiversity on the Tibetan plateau. Anim. Conserv. 2, 235–240. 10.1007/s13280-014-0568-x 25331028 PMC4293360

[B36] Stanton T. Canale-Parola E. (1979). Enumeration and selective isolation of rumen spirochetes. Appl. Environ. Microbiol. 38, 965–973. 543706 10.1128/aem.38.5.965-973.1979 PMC243616

[B37] Tamaki H. Wright C. L. Li X. Lin Q. Hwang C. Wang S.. (2011). Analysis of 16S rRNA amplicon sequencing options on the Roche/454 next-generation titanium sequencing platform. PLoS ONE 6:e25263. 10.1371/journal.pone.0025263 21966473 PMC3179495

[B38] Tung J. Barreiro L. B. Burns M. B. Grenier J. C. Lynch J. Grieneisen L. E.. (2015). Social networks predict gut microbiome composition in wild baboons. eLife 4:e05224. 10.7554/eLife.05224 25774601 PMC4379495

[B39] VanderWaal K. L. Atwill E. R. Isbell L. A. McCowan B. (2014). Linking social and pathogen transmission networks using microbial genetics in giraffe (*Giraffa camelopardalis*). J. Anim. Ecol. 83, 406–414. 10.1111/1365-2656.12137 24117416

[B40] Wang J. Wei W. Zhang Y. Yin B. (2005). Behavior patterns of plateau pika *Ochotona curzoniae* at different population densities. Acta Zool. Sinica 51, 598–607. 10.3321/j.issn:0001-7302.2005.04.007 (In Chinese).

[B41] Wang Q. Garrity G. M. Tiedje J. M. Cole J. R. (2007). Naive Bayesian classifier for rapid assignment of rRNA sequences into the new bacterial taxonomy. Appl. Environ. Microbiol. 73, 5261–5267. 10.1128/AEM.00062-07 17586664 PMC1950982

[B42] Warton D. I. Wright S. T. Wang Y. (2012). Distance-based multivariate analyses confound location and dispersion effects. Methods Ecol. Evol. 3, 89–101. 10.1111/j.2041-210X.2011.00127.x

[B43] Yatsunenko T. Rey F. E. Manary M. J. Trehan I. Dominguez-Bello M. G. Contreras M.. (2012). Human gut microbiome viewed across age and geography. Nature 486, 222–227. 10.1038/nature11053 22699611 PMC3376388

