# Supplementary figures and images for: Pika Population Density Is Associated with the Composition and Diversity of Gut Microbiota
Source: Front Microbiol. 2016 May 18;7:758. doi: 10.3389/fmicb.2016.00758 (PMC4870984; doi:10.3389/fmicb.2016.00758)

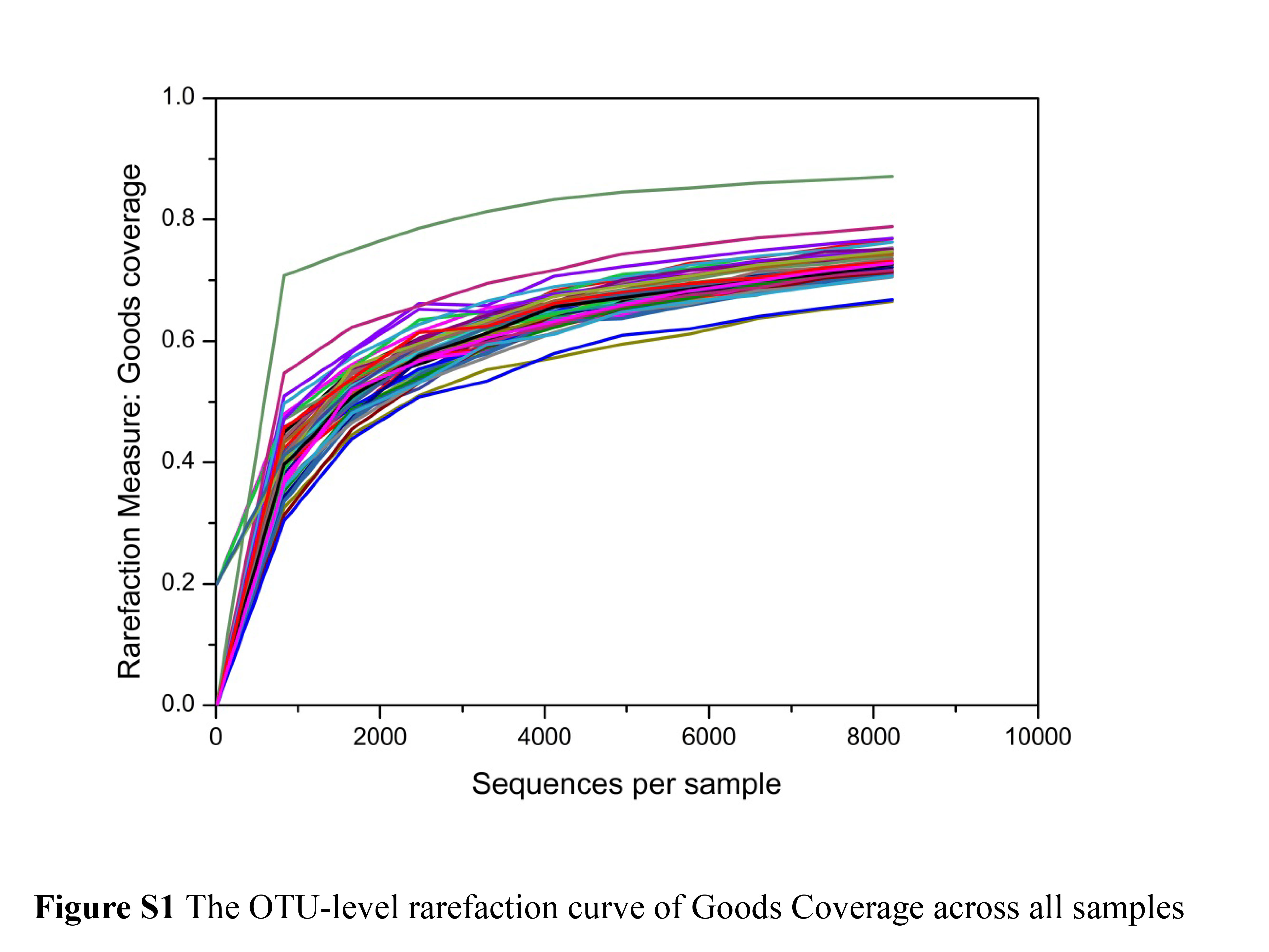

Supplement: Supplementary file 8 [file Image1.JPEG]

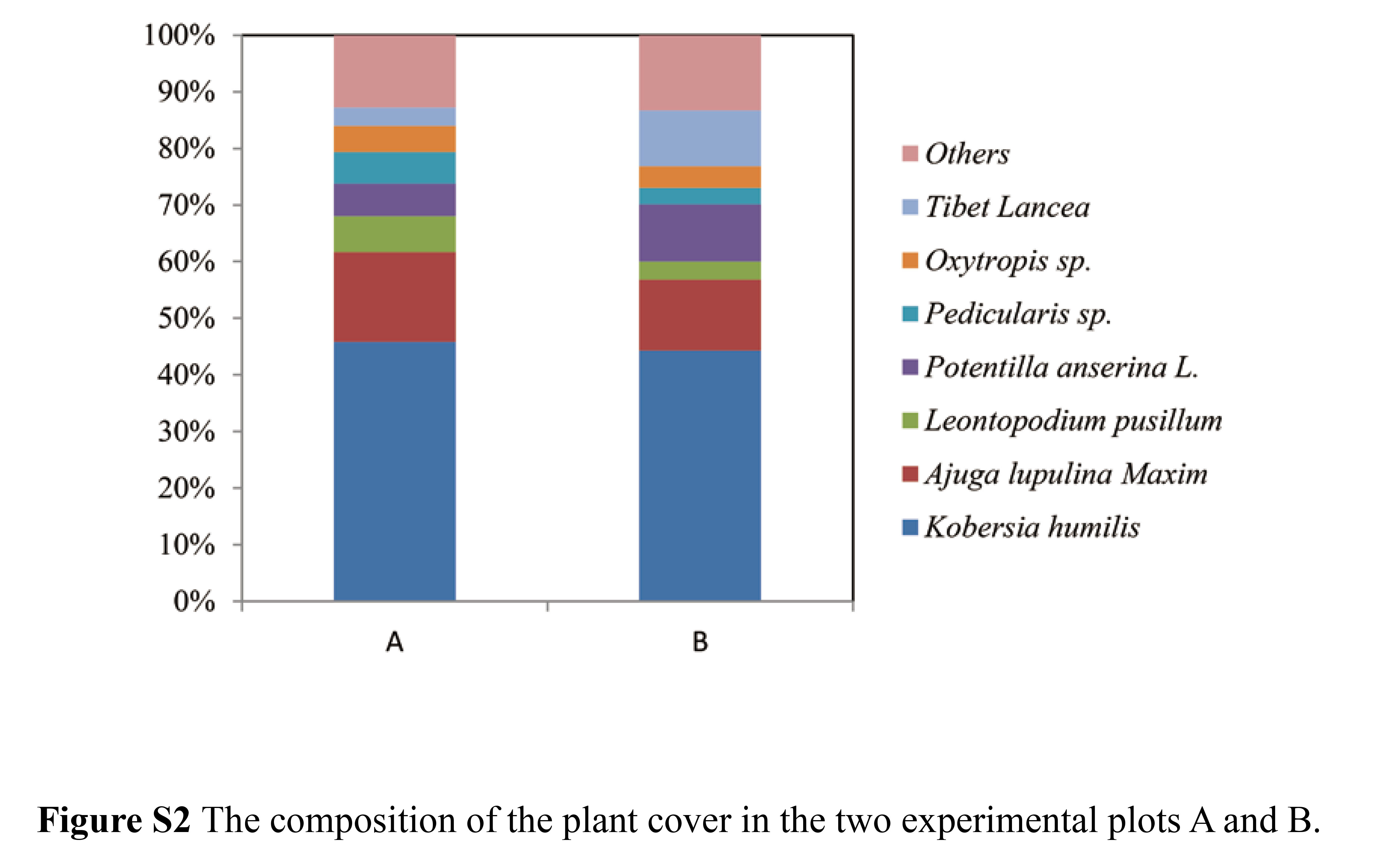

Supplement: Supplementary file 9 [file Image2.JPEG]
